# Dose fractionation of CAR-T cells. A systematic review of clinical outcomes

**DOI:** 10.1186/s13046-022-02540-w

**Published:** 2023-01-10

**Authors:** Matthew Frigault, Anand Rotte, Ayub Ansari, Bradford Gliner, Christopher Heery, Bijal Shah

**Affiliations:** 1 Massachusetts General Hospital Cancer Center, Boston, MA USA; 2 Harvard Medical School, Boston, MA USA; 3 Arcellx Inc, Gaithersburg, MD USA; 4 Moffitt Cancer Center, Tampa, FL USA

**Keywords:** CAR-T cells, Dose, Efficacy, Safety, Tumor burden

## Abstract

CAR-T cells are widely recognized for their potential to successfully treat hematologic cancers and provide durable response. However, severe adverse events such as cytokine release syndrome (CRS) and neurotoxicity are concerning. Our goal is to assess CAR-T cell clinical trial publications to address the question of whether administration of CAR-T cells as dose fractions reduces toxicity without adversely affecting efficacy. Systematic literature review of studies published between January 2010 and May 2022 was performed on PubMed and Embase to search clinical studies that evaluated CAR-T cells for hematologic cancers. Studies published in English were considered. Studies in children (age < 18), solid tumors, bispecific CAR-T cells, and CAR-T cell cocktails were excluded. Data was extracted from the studies that met inclusion and exclusion criteria. Review identified a total of 18 studies that used dose fractionation. Six studies used 2-day dosing schemes and 12 studies used 3-day schemes to administer CAR-T cells. Three studies had both single dose and fractionated dose cohorts. Lower incidence of Grade ≥ 3 CRS and neurotoxicity was seen in fractionated dose cohorts in 2 studies, whereas 1 study reported no difference between single and fractionated dose cohorts. Dose fractionation was mainly recommended for high tumor burden patients. Efficacy of CAR-T cells in fractionated dose was comparable to single dose regimen within the same or historical trial of the same agent in all the studies. The findings suggest that administering dose fractions of CAR-T cells over 2–3 days instead of single dose infusion may mitigate the toxicity of CAR-T cell therapy including CRS and neurotoxicity, especially in patients with high tumor burden. However, controlled studies are likely needed to confirm the benefits of dose fractionation.

## Introduction

Over the last decade, research in cancer immunotherapy has made significant progress in the treatment of cancer and resulted in dramatic improvements in patient survival [1–6]. Chimeric antigen receptor (CAR)-T cells, generated by modifying human autologous or allogeneic T cells to target specific antigens, are shown to have promising response rates in hematologic malignancies and offer durable efficacy [7–10]. There are currently six FDA approved CAR T cell therapies, including axicabtagene ciloleucel (axi-cel), brexucabtagene autoleucel (brexu-cel), tisagenlecleucel (tisa-cel), lisocabtagene maraleucel (liso-cel), idecabtagene vicleucel (ide-cel) and ciltacabtagene autoleucel (cilta-cel) for various types of hematologic cancers including B-cell lymphoma, mantle cell lymphoma, acute lymphoblastic leukemia and multiple myeloma. Though CAR-T cell therapy has been shown to have unique benefits and manageable safety profile unlike that of traditional chemotherapy, the adverse events associated with CAR-T cells such as cytokine release syndrome (CRS) and immune effector cell-associated neurotoxicity syndrome (ICANS) are particularly concerning and are known to be associated with prolonged hospitalizations, increased medical expenditures and even fatalities [11, 12].

While the mechanisms are not completely understood, activation of CAR-T cells is thought to result in the release of IFN-γ, TNF-α, and other inflammatory cytokines that drive macrophage and monocyte release of IL-6 and other cytokines, which may precipitate CRS and ICANS events via endothelial damage [13]. Typically, clinical symptoms of CRS are manifested within 1–7 days of infusion [14] and CAR T cell dose, expansion kinetics of CAR-T cells, and tumor burden are identified as the major factors associated with CRS severity [15, 16]. The symptoms of ICANS can be delayed slightly with time to onset ranging from 2 to 19 days, and CRS has been shown to be a major risk factor associated with severity of ICANS [14, 17–20]. Patients with high tumor burden were shown to have higher risk of CRS in multiple studies [21–24] and severity of CRS was shown to be associated with shorter progression free survival in a retrospective study with relatively small cohort size [25]. Dose fractionation or split dosing of CAR-T cells has been proposed to limit the release of inflammatory cytokines and thereby address the issue of CRS [26]. Administration of dose fractions of CAR-T cells is thought to result in controlled expansion of CAR-T cells and tumor killing and subsequently result in lower peaks of inflammatory cytokines. Indeed, the incidence of Grade 3 or above CRS was as low as ‘0’ in studies that used dose fractionation for the administration of CAR-T cells [27–29].

However, it is not clear if dose fractionation negatively impacts the efficacy of CAR-T cells, and the effects of dose fractionation on incidence and severity of CRS have not been reviewed systematically. The current review aims to evaluate the studies that used dose fractionation to administer CAR-T cells and to discuss the findings from the studies with emphasis on dose fractionation scheme, tumor burden, and efficacy of CAR-T cells.

## Methods

This systematic review followed the guidelines defined by the Preferred Reporting Items for Systematic Reviews and Meta-analyses (PRISMA) Statement [30].

### Search criteria

The following search terms were utilized in the literature search for related articles: “CAR”, “chimeric antigen receptor”, “CAR-T cell”, “acute lymphoblastic leukemia”, “ALL”, “diffuse large B-cell lymphoma”, “DLBCL”, “multiple myeloma” and “MM”. Searches were conducted on PubMed and Embase in May 2022. A total of seven searches were conducted on each database: [1] “CAR” or “chimeric antigen receptor”, [2] “CAR-T cell” and “acute lymphoblastic leukemia” or “ALL”, [3] “CAR-T cell” and “diffuse large B-cell lymphoma” or “DLBCL”, [4] “CAR-T cell” and “multiple myeloma” or CAR” or “MM”, [5] “chimeric antigen receptor” and “acute lymphoblastic leukemia”, [6] “chimeric antigen receptor” and “diffuse large B-cell lymphoma”, and [7] “chimeric antigen receptor” and “multiple myeloma”.

### Eligibility

All clinical prospective and retrospective studies reporting outcomes in adult patients (age ≥ 18 years) with hematologic malignancies including Acute Lymphoblastic Leukemia (ALL), Diffuse Large B-cell Lymphoma (DLBCL) and Multiple Myeloma (MM) met the inclusion criteria for consideration. Studies were excluded if they met any of the following exclusion criteria: [1] Articles reported in languages other than English, [2] Conference presentations and abstracts, [3] Studies that did not use lymphodepletion regimen, [4] Studies in children, [5] Studies in solid tumors, [6] Studies using bispecific CAR-T cells, [7] Studies using CAR-T cell cocktails, [8] Studies using bispecific antibodies, [9] Studies using antibody drug conjugates, [10] Articles reporting additional outcomes/post hoc analyses of previously published study, [11] Preclinical studies, [12] Systematic literature review articles, and [13] Review articles. Bispecific CAR-T cells, solid tumors and studies in children, which are expected to have widely different kinetics, and comparatively different efficacy and safety, are excluded from the review. Studies that fractionated (split) the dose of CAR-T cells and administered over multiple days were identified and were used to answer the questions on dose-fractionation.

### Data extraction

Studies meeting the eligibility criteria were screened based on their title, abstract and full text by two independent reviewers. Reasons for excluding studies were recorded and included studies were cross checked prior to data extraction such that any discrepancy arising between the two reviewers was resolved through discussion. The following data was extracted from each study’s full text: study details (author name and year of publication), patient characteristics (number of patients, cancer subtype, tumor burden), CAR-T cell details (dose and regimen, target antigen and co-stimulatory domains), efficacy outcomes (overall survival, OS; progression free survival, PFS; objective response rate, ORR) and safety outcomes (CRS and neurotoxicity). In order to compare the dose across studies, dose was calculated for 70 kg for studies that used body weight-based dose and for 1.6 m^2^ for studies that used body surface area-based dose and converted to a flat dose value.

## Results

### Characteristics of selected studies

**Fig. 1:**
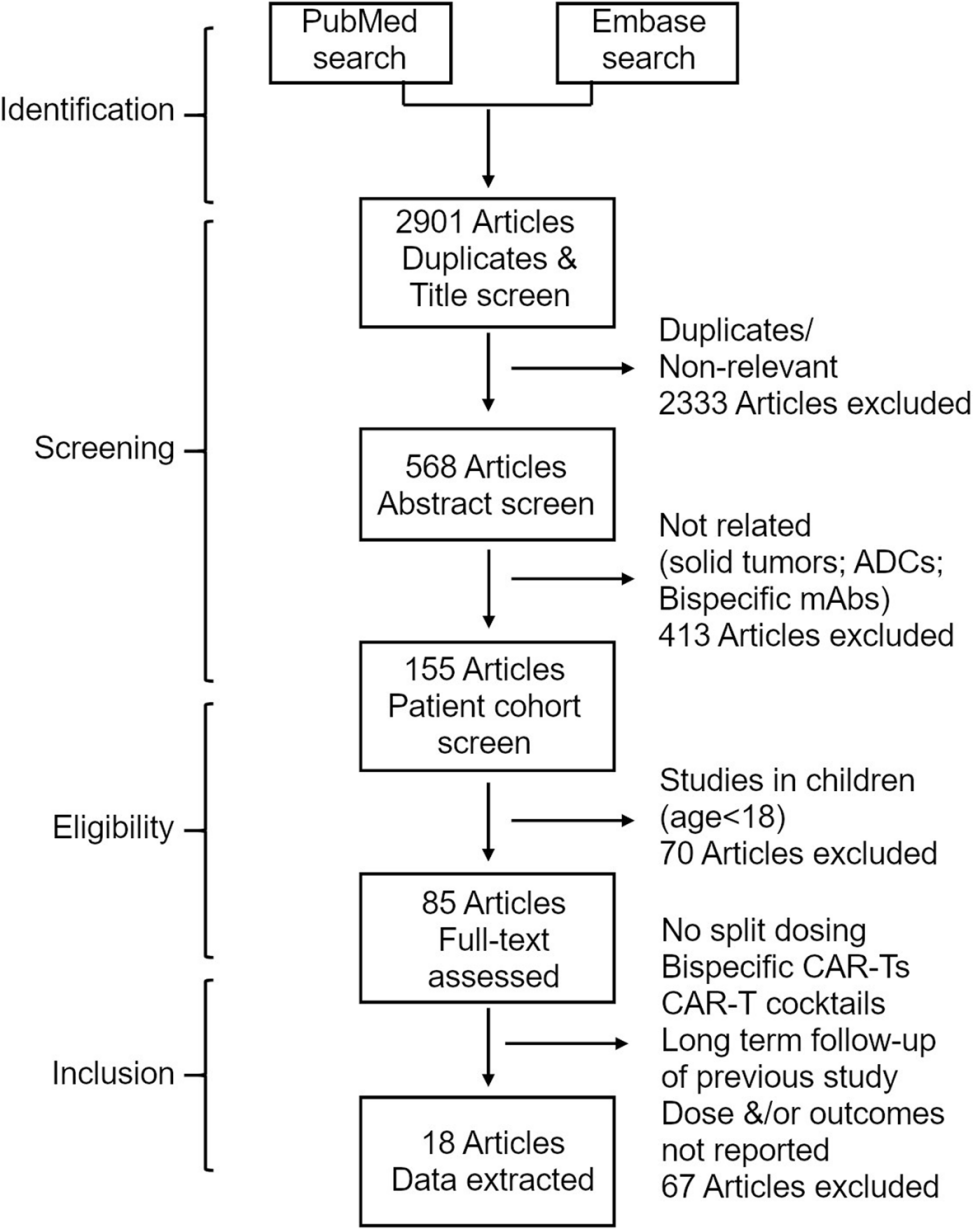
Study flow and selection of articles

Literature search for clinical articles on CAR-T cells published between January 2010 and May 2022 resulted in 2,775 hits. The articles were screened for duplicates and relevance based on title, abstract and then full text by two reviewers. Eighteen articles were identified to meet the inclusion criteria and were selected for data extraction (Fig. 1). Quality assessment was done for the 18 studies using guidelines for non-randomized single-arm studies (Table 1) [31–34]. In total, 430 patients were treated with CAR-T cells and the major indications studied included ALL (n = 196), MM (n = 99) and CLL (n = 73). Three studies also included single dose cohorts in which patients received CAR-T cells in a single dose allowing direct comparison between single dose and fractionated dose groups [35–37]. CAR-T cells were administered over 2 days in 6 studies [27, 29, 38–41] and over 3 days or more in 12 studies [22, 28, 35–37, 42–47]. Administration of CAR-T cells was spread out over consecutive days in all but 2 studies. Roddie et al. administered the second dose of CAR-T cells after 9 days [40] whereas Xu et al. administered 3 dose fractions at an interval of 3 days [36]. Regardless of the interval, none of the studies required additional lymphodepletion regimen before the administration of next dose.

**Table 1:** Quality assessment of included studies

	Risk of bias					Indirectness	Imprecision	
	**Selection bias**	**Attrition bias**	**Reporting/Detection bias**					
**First Author**	**IRC involved in patient selection (Yes; No)**	**Loss to follow-up** **(< 5%; 5–20%; >20%)**	**Objective outcomes assessed (Yes; No)**	**IRC involved in assessment of response (Yes; No)**	**Safety outcomes reported (Yes; No)**	**Heterogeneity** **(Single sub-type; 2 sub-types; >2 sub-types in the study)**	**Sample size** **(< 30; 30–50; >50 patients treated)**	**Duration of follow-up** **(< 6 months; 6–12 months; >12 months)**
**Studies with single dose and fractionated-dose patients**								
Frey NV(37)	No*	> 20%	Yes	No	Yes	Single sub-type	30–50	> 12 months
Frey NV(35)	No*	5–20%	Yes	No	Yes	Single sub-type	30–50	> 12 months
Xu J(36)	No*	Consort Diagram Not Reported	Yes	No	Yes	Single sub-type	< 30	> 12 months
**Anti-CD19 CAR-T cells**								
Li M(48)	No*	> 20%	Yes	No	Yes	Single sub-type	> 50	NR
Jiang H(22)	No*	Consort Diagram Not Reported	Yes	No	Yes	Single sub-type	> 50	NR
Ying Z(28)	No*	Consort Diagram Not Reported	Yes	No	Yes	> 2 sub-types	< 30	NR
Roddie C(40)	No*	Consort Diagram Not Reported	Yes	No	Yes	Single sub-type	< 30	> 12 months
Wang CM(45)	No	Consort Diagram Not Reported	Yes	No	Yes	> 2 sub-types	< 30	NR
Geyer MB(38)	No	> 20%	Yes	No	Yes	> 2 sub-type	< 30	> 12 months
Davila ML(41)	No	< 5%	Yes	No	Yes	Single sub-type	< 30	> 12 months
Sauter CS(39)	No	Consort Diagram Not Reported	Yes	No	Yes	> 2 sub-types	< 30	> 12 months
Hu Y(46)	No	5–20%	Yes	No	Yes	Single sub-type	< 30	< 6 months
Porter DL(47)	No*	> 20%	Yes	No	Yes	Single sub-type	< 30	> 12 months
Geyer MB(27)	No	Consort Diagram Not Reported	Yes	No	Yes	Single sub-type	< 30	> 12 months
Zhang Q(29)	No*	Consort Diagram Not Reported	Yes	No	No	Single sub-type	< 30	NR
Kalos M(44)	No*	Consort Diagram Not Reported	Yes	No	Yes	Single sub-type	< 30	NR
**Anti-BCMA CAR-T cells**								
Zhao WH(43)	No*	Consort Diagram Not Reported	Yes	No	Yes	Single sub-type	> 50	6–12 months
Cohen AD(42)	No	5–20%	Yes	Yes	Yes	Single sub-type	< 30	> 12 months

* Independent review committee/board approved the study’s protocol and had patients sign consent forms

IRC, independent review committee

All observational and single arm unblinded studies are given low grade and the grade is moved upwards based on quality assessment [48–50]

Risk of Bias mainly involves selection bias and reporting or detection bias. Selection bias is low, and quality is high for studies that included an IRC for patient selection and that had < 5% loss of patients to follow-up. Studies with 5–20% loss to follow-up are considered to have medium selection bias and studies with over 20% loss to follow-up are considered to have high selection bias

Reporting or detection bias is considered low for studies that evaluated objective outcomes, included an IRC for response assessment, and reported treatment-related adverse events (safety). Studies that reported subjective outcomes (e.g. patient reported outcomes) or studies that did not include IRC for response assessment or studies that did not report safety outcomes are rated as high for reporting or detection bias

Indirectness (comparability) of the cohort between studies is considered low and quality is also high for studies that have a homogenous cohort (single type of cancer). Studies with up to 2 cancer-subtypes are rated as medium for indirectness and with > 2 cancer-subtypes are rated as low for comparability

Imprecision of the cohort is considered high and quality is low for studies that have low sample size (< 30 patients) and small follow-up (< 6 months). Studies that have a sample size of 30–50 patients or with 6–12 months follow-up are rated medium for imprecision. Studies with sample size of > 50 patients and with follow-up over 12 months are rated low for imprecision and high for quality

### Mitigation of CRS and Neurotoxicity

To address the question on whether dose fractionation helped in reducing the incidence and/or severity of CRS and neurotoxicity, adverse event (AE) data from the studies was collected and the overall conclusion of the authors on the benefits of dose-fractionation was recorded. As seen from the data listed in Table 2, the incidence and/or severity of Grade ≥ 3 CRS and neurotoxicity ranged from 0 to 40% and 0–15% with anti-CD19 CAR-T cells and 7–32% and 0–12% with anti-BCMA CAR-T cells respectively. Authors noted that AEs were mitigated with dose fractionation in most studies [27–29, 35–37, 40, 42, 43, 45, 46]. Intriguingly, there were no Grade ≥ 3 CRS and/or neurotoxicity events following CAR-T cell treatment in studies by Geyer et al., [27] Zhang et al., [29] Roddie et al [40] and Ying et al [28]. However, the overall response rate was also lower in the study by Geyer et al. making it difficult to conclude whether the lower toxicity was indeed due to dose fractionation or due to lower overall activity of the CAR-T product [27]. Similarly, along with dose fractionation, low affinity binder was used in the study by Roddie et al. and a variant of CD19-BBz CAR that showed lower cytokine production and comparable cytotoxic ability in the in vitro experiments was used in the study by Ying et al. to address CRS confounding the interpretation of the results [28, 40].

**Table 2:** Grade ≥ 3 CRS and ICANS, and response rates

Study details (Author; Indication)	N	Dose/s used (million cells)^a^	Dose and Fractionation regimen (% of total dose)	Grade ≥ 3 CRS and ICANS	Response Rate
**Studies with single dose and fractionated-dose patients**					
Frey NV(35); ALL	FD, 20; SD, 6	500	10, 30, 60 (d1,2,3)	CRS, 5% in FD patients and 50% SD patients ICANS not reported	CRR, 90% in FD patients and 50% in SD patients ORR not reported
Frey NV(37); CLL	FD, 8; SD, 11	500	10, 30, 60 (d1,2,3)	CRS, 25%; ICANS, 0% in FD patients CRS, 36%; ICANS, 18% in SD patients	ORR, 50%; CRR, 38% in FD patients ORR, 55%; CRR, 36% in SD patients
Xu J(36); Multiple Myeloma	FD, 8; SD, 9	49 (average)	0, 3, 6d (33% each)	CRS, 63% in FD patients and 22% in SD patients	ORR, 100%; CRR, 71% in FD patients ORR, 89%; CRR, 89% in SD patients
**Anti CD19 CAR-T cells**					
Li M(48); B-ALL	78	350	10, 30, 60	CRS, 29%; ICANS, not reported	ORR/CRR, 83%
Jiang H(22); B-ALL	53	280.7	No details (0-2d)	CRS, 36%; Grade 2/3 ICANS, 15%	ORR/CRR, 89%
Ying Z(28); B-cell lymphoma	25	DL1, 3–6; DL2 60–190; DL3, 200–400	30, 30, 40 (0-2d)	CRS, 0%; ICANS, 0%	ORR, 60%; CRR, 28%
Roddie C(40); B-ALL	20	410	33, 67 or 2.5, 97.5 on d0, d9	CRS, 0%; ICANS, 15%	ORR/CRR, 85%
Wang CM(45); Hodgkins Lymphoma	18	770–1470	starting with 21 M, increments of 5-fold (e.g. 100 M cells) over 3–5 days	CRS and ICANS, 0%	ORR, 39%; CRR, 0%
Geyer MB(38); CLL	16	280–2100	33, 67 (0,1d)	CRS, 13%; ICANS, 6%	ORR/CRR, 19%
Davila ML(41); B-ALL	16	210	33, 67 d1, d2	Severe CRS, 44% and severe ICANS, 25% (not categorized as Grade 3+)	ORR/CRR, 88%
Sauter CS(39); B-cell Lymphoma	15	DL1, 350; DL2, 700	67, 33 (d2, d3)	CRS, 20%; ICANS, 6%	ORR/CRR, 53%
Hu Y(46); ALL	15	77–686	No details (0-2d)	CRS, 40%; ICANS, not reported	ORR/CRR, 73%
Porter DL(47); CLL	14	14-1100	10, 30, 60 (0-2d)	CRS, 43%; ICANS, 7%	ORR, 57%; CRR, 29%
Geyer MB(27); CLL	8	280–2100	33, 67 (0,1d)	CRS, 0%; ICANS, 0%	ORR/CRR, 25%
Zhang Q(29); B-ALL	4	ND	33, 67 (2d)	CRS, 0%; ICANS, not reported	ORR, 75%; CRR, not reported
Kalos M(44); CLL	3	140, 580 and 1100	10, 30, 60 (0-2d)	Not reported	ORR, 100%; CRR, 67%
**Anti-BCMA CAR-T cells**					
Zhao WH	57	4.9 to 147	20%, 30%, 50% (0-7d)	CRS, 7%; ICANS, 0%	ORR, 88%; CRR, 68%
Cohen AD	25	10–500	10, 30, 60 (0-2d)	CRS, 32%; ICANS, 12%	ORR, 48%; CRR, 8%

*dose calculated for 70 kg if administered per kg

Within the studies that also included a single dose cohort, two studies were from same team of investigators and evaluated anti-CD19 CAR-T cells whereas the 3rd study evaluated anti-BCMA CAR-T cells (Table 2) [35–37]. Interestingly, both anti-CD19 CAR-T cell studies noted that dose fractionation reduced the toxicity of CAR-T cells whereas the anti-BCMA CAR-T cell study reported that CRS severity was similar between single dose and fractionated dose groups.

In the study in adults with relapsed/refractory ALL, Frey et al. assigned patients to 3 dosing cohorts including low dose (n = 9), high dose (single; n = 6) and high dose (fractionated; n = 20) groups depending on time of enrollment. Patients in the high dose groups received CTL-09 (tisa-cel) at 500 million cells as single or fractionated dose split over 3 days (10%, 30%, 60%) depending on their treatment plan. The study reported that the incidence of severe Grade 4/5 CRS was significantly lower (Fischer exact test; p = 0.017) in the high dose fractionated group (5% vs. 22% and 50%) compared to low single dose and high single dose groups [35]. In the second study, patients with CLL received CART-19 (tisa-cel) cells either as single dose (stage I) at 50 or 500 million cells or fractionated dose (stage II) at 500 million cells. The findings from the study showed that the incidence of Grade ≥ 3 CRS (25% vs. 36%, respectively) and ICANS (0% vs. 18%, respectively) were lower in the fractionated dose group compared to the single dose group [37].

In the third study by Xu, et al., patients with relapsed/refractory multiple myeloma were enrolled in two dose cohorts including single infusion (n = 8) and fractionated infusion (n = 9) [36]. Patients received LCARB38M (cilta-cel) at 14 to 140 million cells (for 70 kg patient; median 49 million cells) as a single dose or as 3 equal fractions administered on days 0, 3 and 6 depending on the hospital of treatment. Patients treated at Ruijin hospital (identified as RJ in the study) and at Changzheng hospital (identified as CZ in the study) received split infusion whereas patients at the First Affiliated Hospital of Nanjing Medical University (identified as JS in the study) received single infusion. Authors concluded that there were no obvious efficacy and toxicity differences between fractionated dose and single dose sub-groups. Interestingly, the incidence of Grade ≥ 3 CRS appeared to be comparatively higher in the fractionated dose group in the Xu et al. study [36], but the incidence was much lower (CRS, 7%; ICANS, 0%) in another study [43] evaluating the same CAR-T cell therapy (LCARB38M/cilta-cel) using fractionated dosing.

### Tumor burden

Toxicity of CAR-T cells has been shown to correlate with tumor burden in clinical studies and the incidence of severe CRS (Grade ≥ 3) was shown to be high in patients with high tumor burden [51–54]. Risk adaptive dose reduction of CAR-T cells has been proposed as a strategy in patients with high tumor burden [54]. Understandably, high tumor burden was cited as one of the main reasons for evaluating dose fractionation of CAR-T cells in all the studies. Moreover, dose fractionation design was also intended to help in stopping infusion of second/next fraction of the dose if the patient had an intolerable CRS event [40]. However, the definition of ‘high’ tumor burden varied widely across the studies (5–50% blasts in B-ALL and CLL; 20–50% plasma cells in MM) and details of tumor burden was provided only in 8 studies [22, 27, 29, 35, 36, 40–42, 46, 47]. The incidence of Grade ≥ 3 CRS ranged from 0 to 44% in the studies that provided the tumor burden information. The studies by Davila et al [41] and Porter et al [47] had relatively higher percentage of patients with ‘high’ tumor burden (6/15 patients with ≥ 50% blasts and median blasts 88% respectively) and the incidence of Grade ≥ 3 CRS was accordingly higher (> 40%), but authors of both studies opined that dose fractionation helped in reducing the severity of CRS.

### Efficacy of CAR-T cells

One of the concerns with using fractionated doses of CAR-T cells is the possibility of reduced efficacy. Administration of lower doses of CAR-T cells initially followed by higher doses may theoretically impact in vivo expansion of CAR-T cells and subsequently the efficacy of CAR-T cells. To answer the question on whether the efficacy of CAR-T cells is reduced in patients receiving dose fractions of CAR-T cells, response rates of the studies were extracted and compared to historical rates. Similarly, efficacy was compared between single dose and fractionated dose groups in the Frey et al. and Xu et al. studies [35, 36]. As seen in Table 2 the response rates in patients receiving fractionated doses were comparable to the historical responses to single dose administration [55]. The dose fractionated group in Frey et al. study had significantly higher complete response (CR) rate (90% vs. 50% respectively; p = 0.0038), longer event-free survival (EFS; 19.4 months vs. 2.4 months respectively; p = 0.0003) and longer overall survival (OS; not reached vs. 3.4 months respectively; p = 0.003) compared to single dose group. The 2-year OS (73% vs. 17% respectively) and EFS (50% vs. 17% respectively) rates were higher in the dose fractionated group compared to single dose group [35]. The ORR in the Xu et al. study was comparable between dose fractionated group (7/8; 1 not evaluable) and single dose group (8/9; 1 minimal response), but the stringent CR (sCR) rate was comparatively higher in the single dose group (5/7 sCR, 2/7 very good partial response and 8/8 sCR respectively). However, 5/8 patients in the single dose group had progressive disease compared to 2/7 patients in fractionated dose group and the duration of response also tended to be longer in the fractionated dose group [36].

## Discussion

The current systematic review aimed to compile the studies that used dose-fractionation of CAR-T cells and summarize the efficacy and safety outcomes, which was not addressed in previous systematic reviews on CAR-T cells [55–62]. In planning this review, studies were expected to be inconsistent in reporting the tumor burden and the cut-off for ‘high’ tumor burden was expected to vary across the studies. Wide dose ranges were expected to be used across the studies and the CAR-T cell product efficiency was expected to vary significantly across the studies. Combining the data from all the studies was expected to average the incidence and severity of adverse events in favor of CAR-T cell products with low efficacy and toxicity and/or in favor of patients with low tumor burden [27]. Therefore, the review did not intend to pool the efficacy or safety data from the studies and analyze the pooled data but assessed each study separately based on the outcomes reported and derived independent conclusions on efficacy and safety improvements or lack of improvements with dose-fractionation. Similarly, the efficacy and safety profile of CAR-T cells in pediatric patients and young adults was expected to be different from adults and were considered for exclusion. Interestingly, none of the studies in children that were identified during literature search used dose fractionation for administration of CAR-T cells. The studies that had parallel comparisons between single dose and dose-fractionation groups were discussed in detail [35–37].

CRS and neurotoxicity following CAR-T cell treatment are serious adverse events resulting in prolonged hospitalization and in some cases fatal consequences [63, 64]. In addition, CRS and neurotoxicity have been shown to be a risk factor for development of infections and related mortality in patients treated with CAR-T cells [65, 66]. While management of CRS and neurotoxicity is less of a concern based on improved toxicity management algorithms, including the use of dexamethasone and tocilizumab [26, 54, 67, 68], patients with high tumor burden and CAR-T cell therapies requiring higher doses (> 150 million cells) could be at a greater risk of toxicity. Therefore, strategies to prevent the development of CRS are needed.

**Fig. 2:**
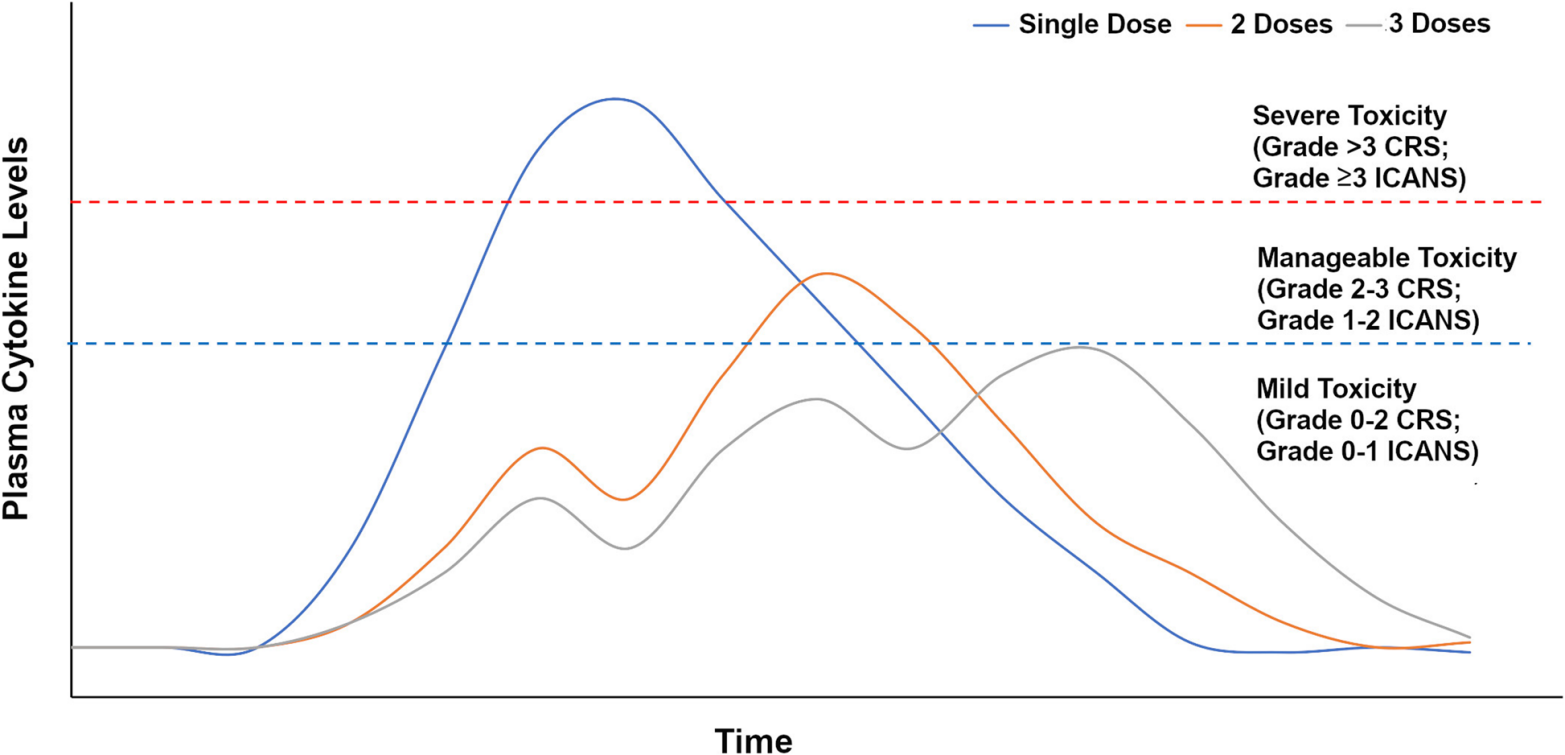
Model showing cytokine levels and toxicity with single dose and fractionated dose. At a constant dose and tumor burden (high), the administration of CAR-T cells as a single infusion can result in the peak cytokine levels that correlate with severe CRS whereas administration of CAR-T cells as 2 or 3 doses can result in lower cytokine levels that correlate with mild-to-moderate CRS.

Administration of CAR-T cells as dose fractions [35, 40] instead of single dose has been proposed as a strategy based on the association between CRS severity, inflammatory cytokine peak following CAR-T cell administration and tumor burden. Severity of CRS has been shown to correlate with peak levels of inflammatory cytokines (cytokine peak) such as IL-6 in patients treated with CAR-T cells [69]. In turn, cytokine peak was shown to correlate with tumor burden [51] and with CAR-T cell dose [69]. Thus, at a constant dose, tumor burden correlates with the severity of CRS, and in patients with high tumor burden, lowering the dose could reduce the peak cytokine levels and the severity of CRS. However, lowering the dose may also result in inadequate effector to tumor cell ratio, resulting in incomplete clearance of all tumor cells. The result is a need to distribute the expansion of a relatively high CAR T cell dose over time. Therefore, administration of CAR-T cells as dose fractions over 2–3 days could stagger the rise in cytokine levels thereby resulting in lower peaks and consequently lower the severity of CRS (Fig. 2). The findings from this review suggests that there is limited clinical trial evidence supporting the hypothesis that administration of CAR-T cells as dose fractions instead of single dose may reduce the severity of CRS. Additional studies are needed to conclusively confirm the benefits of dose fractionation. CAR-T cell expansion data reported in Xu et al. study showed that though the expansion peak tended to be higher and time to peak appeared to be earlier with single dose, other pharmacokinetic parameters of CAR-T cell kinetics such as area under the curve and persistence were similar in the patients receiving single dose infusion and fractionated dose infusion [36]. Finally the results from the included studies showed that efficacy of CAR-T cells in the patients who received fractionated dose was comparable to single dose administration.

Dose fractionation mainly has the additional advantage of flexibility to stop the treatment when severe CRS is developed in the patient following infusion of first dose fraction and ability to modify the overall dose depending on product characteristics. Frey et al. used 3-day dosing regimen in which second and/or third dose of CAR-T cells were withheld if patients showed early signs of CRS including fever [35]. In another dosing scheme, Roddie et al. divided the enrolled ALL patients based on tumor burden into two groups of CAR-T cell dosing regimen. Patients with > 20% blasts were infused with 10 million CAR-T cells and patients with ≤ 20% blasts received 100 million CAR-T cells on the day of infusion. Patients received the second dose fraction of CAR-T cells (400 million and 310 million cells respectively) after 9 days if there were no Grade ≥ 3 CRS or ICANS or unresolved Grade 1–2 ICANS [40]. Additional studies with larger cohort size could help in identifying the optimal scheme for dose fractionation of CAR-T cells.

The current review did not aim to address the question on total dose of CAR-T cells needed for the treatment. The question was addressed in other studies including our own unpublished systematic review on dose-response association [14, 70–73]. Several factors including target antigen, intracellular signaling domain, and product characteristics such as cell viability and number of CAR molecules per cell, may impact the efficacy and toxicity of the drug product. All of these factors should be considered in determining the total dose of CAR-T cells. The dose that achieves an optimal effector (CAR-T cell)-to-tumor target cell (E:T) ratio may result in the maximal eradication of the tumor cells and achieve the most durable responses for a given product. Lower doses may result in sub-optimal E:T ratio and exhaustion of CAR-T cells whereas very high doses may precipitate intolerable toxicity. To determine the optimal dose needed to achieve optimal E:T ratio, researchers and clinicians may use the data from clinical studies in similar target antigen, indication, and tumor burden as well as the approved label dose for commercially available drug products.

### Limitations

All the studies included in this review lack control group, are non-randomized and open label and the majority had small sample size. Majority of the studies did not include independent review committee for selection of subjects (potential selection bias) and had > 20% loss of subjects to follow-up (potential attrition bias; Table 1). Furthermore, excepting studies by Frey et al. and Xu et al., there was no direct comparison between single dose and dose fractionation.

## Conclusion

In summary, current findings suggest that administering dose fractions of CAR-T cells over 2–3 days instead of single dose infusion may mitigate the incidence and/or severity of CAR-T cell toxicity including CRS and neurotoxicity, especially in patients with high tumor burden and for CAR-T cell therapies requiring higher doses for efficacy. Data further indicate that the effectiveness of the therapy is not adversely affected by dose fractionation. However, the evidence in favor of dose fractionation is limited and additional studies are needed to confirm the benefits of dose fractionation.

## Data Availability

All the data generated is included within the manuscript and its supplementary files.
